# GSVA: gene set variation analysis for microarray and RNA-Seq data

**DOI:** 10.1186/1471-2105-14-7

**Published:** 2013-01-16

**Authors:** Sonja Hänzelmann, Robert Castelo, Justin Guinney

**Affiliations:** 1 Research Program on Biomedical Informatics (GRIB), Hospital del Mar Medical Research Institute (IMIM), Barcelona, Catalonia, Spain; 2 Department of Experimental and Health Sciences, Universitat Pompeu Fabra, Barcelona, Catalonia, Spain; 3 Sage Bionetworks, 1100 Fairview Ave N., Seattle, Washington, 98109, USA

## Abstract

**Background:**

Gene set enrichment (GSE) analysis is a popular framework for condensing information from gene expression profiles into a pathway or signature summary. The strengths of this approach over single gene analysis include noise and dimension reduction, as well as greater biological interpretability. As molecular profiling experiments move beyond simple case-control studies, robust and flexible GSE methodologies are needed that can model pathway activity within highly heterogeneous data sets.

**Results:**

To address this challenge, we introduce Gene Set Variation Analysis (GSVA), a GSE method that estimates variation of pathway activity over a sample population in an unsupervised manner. We demonstrate the robustness of GSVA in a comparison with current state of the art sample-wise enrichment methods. Further, we provide examples of its utility in differential pathway activity and survival analysis. Lastly, we show how GSVA works analogously with data from both microarray and RNA-seq experiments.

**Conclusions:**

GSVA provides increased power to detect subtle pathway activity changes over a sample population in comparison to corresponding methods. While GSE methods are generally regarded as end points of a bioinformatic analysis, GSVA constitutes a starting point to build pathway-centric models of biology. Moreover, GSVA contributes to the current need of GSE methods for RNA-seq data. GSVA is an open source software package for R which forms part of the Bioconductor project and can be downloaded at http://www.bioconductor.org.

## Background

The ability to measure mRNA abundance at a genomic scale has led to many efforts to catalog the diverse molecular patterns underlying biological processes. To facilitate the interpretation and organization of long lists of genes resulting from microarray experiments, gene set enrichment (GSE) methods have been introduced. They systematically measure and annotate molecular profiles that are inherently noisy and difficult to interpret. GSE analyses begin by obtaining a ranked gene list, typically derived from a microarray experiment that studies gene expression changes between two groups. The genes are then mapped into predefined gene sets and their gene expression statistic is summarized into a single enrichment score for each gene set. A significant benefit of these pathway-based methods is interpretability: gene function is collectively exerted and may vary by environmental stimuli, genetic modifications, or disease state. Thus, organizing genes into gene sets provides a more intuitive and stable context for assessing biological activity.

Many methodological variations of GSE methods have been proposed [1-6], including non-parametric enrichment statistics [4,7], battery testing [8-10], and focused gene set testing [1,11,12]. Battery testing methods aim at identifying gene sets standing out from a large collection of annotated pathways and gene signatures. Focused gene set testing methods try to carefully evaluate a few gene sets that are relevant to the experiment being analyzed [12]. GSE methods have been successfully applied in many experimental conditions to interpret the pathway architecture of biological states including cancer [13,14], metabolic disease [15], and development [16]. For a recent review on GSE methods the reader may consult [17].

An important distinction among many of the GSE methods is the definition of the null hypothesis that is tested [18]. The null hypothesis of a competitive test declares that there are no differences between genes inside and outside the gene set (e.g., [4]). A self-contained test defines its null hypothesis only in terms of the genes inside the gene set being tested (e.g., [1]). More concretely, for a self-contained test on a gene set, the differential expression of just one of its genes allows one to reject the null hypothesis of no differential expression for that gene set. It follows, that self-contained tests provide higher power than competitive tests to detect subtle changes of expression in a gene set. But they may not be useful to single out a few gene sets in a battery testing setting because of the potentially large number of reported results.

Finally, many GSE methods assume two classes (e.g. case/control) and evaluate enrichment within this context [19-22]. The limits imposed by this assumption become evident with the rise of large genomic studies, such as The Cancer Genome Atlas project (TCGA - http://cancergenome.nih.gov), an ambitious project with the goal to identify the molecular determinants of multiple cancer types. In contrast to case-control studies with small sample sizes, the TCGA project has large patient cohorts with multiple phenotypes, structured with hierarchical, multi-class, and censored data. Hence, GSE methods are needed that can assess pathway variation across large, heterogeneous populations with complex phenotypic traits.

To address these challenges, we present a non-parametric, unsupervised method called Gene Set Variation Analysis (GSVA). GSVA calculates sample-wise gene set enrichment scores as a function of genes inside and outside the gene set, analogously to a competitive gene set test. Further, it estimates variation of gene set enrichment over the samples independently of any class label. Conceptually, this methodology can be understood as a change in coordinate systems for gene expression data, from *genes* to *gene sets*. This transformation facilitates post-hoc construction of pathway-centric models, such as differential pathway activity identification or survival prediction. Further, we demonstrate the flexibility of GSVA by applying it to RNA-seq data.

## Implementation

**Figure 1:**
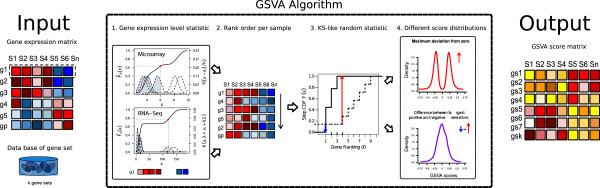
**GSVA methods outline.** The input for the GSVA algorithm are a gene expression matrix in the form of log2 microarray expression values or RNA-seq counts and a database of gene sets. 1. Kernel estimation of the cumulative density function (kcdf). The two plots show two simulated expression profiles mimicking 6 samples from microarray and RNA-seq data. The *x*-axis corresponds to expression values where each gene is lowly expressed in the four samples with lower values and highly expressed in the other two. The scale of the kcdf is on the left *y*-axis and the scale of the Gaussian and Poisson kernels is on the right *y*-axis. 2. The expression-level statistic is rank ordered for each sample. 3. For every gene set, the Kolmogorov-Smirnov-like rank statistic is calculated. The plot illustrates a gene set consisting of 3 genes out of a total number of 10 with the sample-wise calculation of genes inside and outside of the gene set. 4. The GSVA enrichment score is either the maximum deviation from zero (top) or the difference between the two sums (bottom). The two plots show two simulations of the resulting scores under the null hypothesis of no gene expression change (see main text). The output of the algorithm is a matrix containing pathway enrichment scores for each gene set and sample.

A schematic overview of the GSVA method is provided in Figure 1, which shows the two main required inputs: a matrix *X*={*x*_*ij*_}_*p*×*n*_ of normalized expression values (see Methods for details on the preprocessing steps) for *p* genes by *n* samples, where typically *p*≫*n*, and a collection of gene sets $\Gamma =\{\gamma_{1},\ldots ,\gamma_{m}\}$. We shall denote by *x*_*i*_ the expression profile of the *i*-th gene, by *x*_*ij*_ the specific expression value of the *i*-th gene in the *j*-th sample, and by *γ*_*k*_ the subset of row indices in *X* such that *γ*_*k*_⊂{1,… *p*} defines a set of genes forming a pathway or some other functional unit. Let |*γ*_*k*_| be the number of genes in *γ*_*k*_.

GSVA starts by evaluating whether a gene *i* is highly or lowly expressed in sample *j* in the context of the sample population distribution. Probe effects can alter hybridization intensities in microarray data such that expression values can greatly differ between two non-expressed genes [23]. Analogous gene-specific biases, such as GC content or gene length have been described in RNA-seq data [24]. To bring distinct expression profiles to a common scale, an expression-level statistic is calculated as follows. For each gene expression profile $x_{i}=\{x_{i1},\ldots ,x_{\text{in}}\}$, a non-parametric kernel estimation of its cumulative density function is performed. In the case of microarray data, a Gaussian kernel ( [25], pg. 148) is used:

(1) $${\hat{F}}_{h_{i}}(x_{\text{ij}})=\frac{1}{n}\overset{n}{\underset{k=1}{\sum }}\int_{-\infty }^{\frac{x_{\text{ij}}-x_{\text{ik}}}{h_{i}}}\frac{1}{\sqrt{2\Pi }}e^{-\frac{t^{2}}{2}}\text{dt}\;,$$

where *h*_*i*_ is the gene-specific bandwidth parameter that controls the resolution of the kernel estimation, which is set to *h*_*i*_=*s*_*i*_/4, where *s*_*i*_ is the sample standard deviation of the *i*-th gene (Figure 1, step 1). In the case of RNA-seq data, a discrete Poisson kernel [26] is employed:

(2) $${\hat{F}}_{r}(x_{\text{ij}})=\frac{1}{n}\overset{n}{\underset{k=1}{\sum }}\overset{x_{\text{ij}}}{\underset{y=0}{\sum }}\frac{e^{-(x_{\text{ik}}+r)}{\left(x_{\text{ik}}+r\right)}^{y}}{y!}\;,$$

where *r*=0.5 in order to set the mode of the Poisson kernel at each *x*_*ik*_, because the mode of a Poisson distribution with an integer mean *λ* occurs at *λ* and *λ*−1, and at the largest integer smaller than *λ* when *λ* is continuous.

Let *z*_*ij*_ denote the previous expression-level statistic ${\hat{F}}_{h_{i}}(x_{\text{ij}})$, or ${\hat{F}}_{r}(x_{\text{ij}})$, depending on whether *x*_*ij*_ are continuous microarray, or discrete count RNA-seq values, respectively. The following step condenses expression-level statistics into gene sets by calculating sample-wise enrichment scores. To reduce the influence of potential outliers, we first convert *z*_*ij*_ to ranks *z*_(*i*)*j*_ for each sample *j* and normalize further *r*_*ij*_=|*p*/2−*z*_(*i*)*j*_| to make the ranks symmetric around zero (Figure 1, step 2). This is done to up-weight the two tails of the rank distribution when computing the final enrichment score.

We assess the enrichment score similar to the GSEA and ASSESS methods [4,27] using the Kolmogorov-Smirnov (KS) like random walk statistic (Figure 1, step 3):

(3) $$\nu_{\text{jk}}(\ell )=\frac{\overset{\ell }{\underset{i=1}{\sum }}|r_{\text{ij}}{|}^{\tau }I(g_{\left(i\right)}\in \gamma_{k})}{\overset{p}{\underset{i=1}{\sum }}|r_{\text{ij}}{|}^{\tau }I(g_{\left(i\right)}\in \gamma_{k})}-\frac{\overset{\ell }{\underset{i=1}{\sum }}I(g_{\left(i\right)}\notin \gamma_{k})}{p-|\gamma_{k}|},$$

where *τ* is a parameter describing the weight of the tail in the random walk (default *τ*=1), *γ*_*k*_ is the *k*-th gene set, *I*(*g*_(*i*)_∈*γ*_*k*_) is the indicator function on whether the *i*-th gene (the gene corresponding to the *i*-th ranked expression-level statistic) belongs to gene set *γ*_*k*_, |*γ*_*k*_| is the number of genes in the *k*-th gene set, and *p* is the number of genes in the data set. Conceptually, Eq. 3 produces a distribution over the genes to assess if the genes in the gene set are more likely to be found at either tail of the rank distribution (see [4,27] for a more detailed description).

We offer two approaches for turning the KS like random walk statistic into an enrichment statistic (ES) (also called GSVA score), the classical maximum deviation method [4,27,28] and a normalized ES. The first ES is the maximum deviation from zero of the random walk of the *j*-th sample with respect to the *k*-th gene set:

(4) $$ES_{\text{jk}}^{\text{max}}=\nu_{\text{jk}}[\arg\;\underset{\ell =1,\ldots ,p}{\max}\left|\nu_{\text{jk}}(\ell )\right|].$$

For each gene set *k*, this approach produces a distribution of enrichment scores that is bimodal (Figure 1, step 4, top panel, Additional file 1: Figure S1). This is an intrinsic property of the KS like random walk, which generates non-zero maximum deviations under the null distribution. In GSEA [4] it is also observed that the empirical null distribution obtained by permuting sample labels is bimodal and, for this reason, significance is determined independently using the positive and negative sides of the null distribution. In our case, we would like to provide a standard Gaussian distribution of enrichment scores under the null hypothesis of no change in pathway activity throughout the sample population. For this purpose we propose a second, alternative score that produces an ES distribution approximating this requirement (Figure 1, step 4, bottom panel, Additional file 1: Figure S1):

(5) $$\;ES_{\text{jk}}^{\text{diff}}\;=\;\left|ES_{\text{jk}}^{+}\right|-\left|ES_{\text{jk}}^{-}\right|\;=\;\underset{\ell =1,\ldots ,p}{\max}(0,\nu_{\text{jk}}(\ell ))-\underset{\ell =1,\ldots ,p}{\min}(0,\nu_{\text{jk}}(\ell ))\;,$$

where $ES_{\text{jk}}^{+}$ and $ES_{\text{jk}}^{-}$ are the largest positive and negative random walk deviations from zero, respectively, for sample *j* and gene set *k*. This statistic may be compared to the Kuiper test statistic [29], which sums the maximum and minimum deviations to make the test statistic more sensitive in the tails. In contrast, our test statistic penalizes deviations that are large in both tails, and provides a “normalization” of the enrichment score by subtracting potential noise. There is a clear biological interpretation of this statistic, it emphasizes genes in pathways that are concordantly activated in one direction only, either over-expressed or under-expressed relative to the overall population. For pathways containing genes strongly acting in both directions, the deviations will cancel each other out and show little or no enrichment. Because this statistic is unimodal and approximately normal (as observed via simulation, see below), downstream analyses which may impose distributional assumptions on the data are thus possible. In certain cases, the characteristics of this statistic may be undesirable, especially if the relevant gene sets are not explicitly separated into “up” and “down” behavior (as the MSigDB provides for many gene sets). In such circumstances, the statistic defined by Eq. 4 should be used.

Figure 1, step 4 and Additional file 1: Figure S1 show a simple simulation where standard Gaussian deviates are independently sampled from *p*=20,000 genes and *n*=30 samples, thus mimicking a null distribution of no change in gene expression. One hundred gene sets are uniformly sampled at random from the *p* genes with sizes ranging from 10 to 100 genes. Using these two inputs, we calculate the maximum deviation ES and the normalized ES. The resulting distributions are depicted in Figure 1, step 4 and Additional file 1: Figure S1.

Although the GSVA algorithm itself does not evaluate statistical significance for the enrichment of gene sets, significance with respect to a phenotype can be easily evaluated using conventional statistical models. Likewise, false discovery rates can be estimated by permuting the sample labels (Methods). We make no general prescription for thresholds of significance or false discovery, as these choices are highly context dependent and may vary according to each experiment. Examples of these techniques are provided in the following section.

## Results

### Review of other methods

Methods for gene set enrichment can be generally partitioned according to the criteria of supervised vs unsupervised, and population vs single sample assessments. Most GSE methods, such as GSEA [4], are supervised and population based, in that they compute an enrichment score per gene set to describe the entire data set, modeled on a phenotype (discrete, such as case-control, or continuous). The simplest of this genre is described by Tian *et al.*[6,19], evaluated as the mean differential expression (e.g. case vs control) of a set of genes, compared to those genes not in the gene set. One of the major drawbacks of this method is that gene correlations are not taken into account, which might lead to an increased number of false-positive gene sets with respect to GSEA [30]. Many other supervised, population based approaches have also been described [12,17,20,31-34].

A supervised, single sample based approach was introduced in the ASSESS method [27]. After dichotomizing the samples based on phenotypic classes, the ASSESS method computes density estimates for each gene/class followed by the evaluation of an enrichment score for each sample/gene set. This method is well-suited for assessing gene set variation across a dichotomous phenotype. GSVA also utilizes density estimates for evaluating sample-wise enrichment, but by omitting phenotypic information, it enables more general downstream analyses and therefore broader applications.

Three unsupervised, single sample enrichment methods have been developed, Pathway Level analysis of Gene Expression (PLAGE), single sample GSEA (ssGSEA) and the combined z-score [5,22,35]. These methods compute an enrichment score for each gene set and individual sample. PLAGE standardizes each gene expression profile over the samples and then estimates the pathway activity profiles for each gene set as the coefficients of the first right-singular vector of the singular value decomposition of the gene set ( [35], pg. 9). The combined z-score method [22] standardizes first, as PLAGE, each gene expression profile into z-scores but the pathway activity profile is then obtained by combining the individual gene z-scores per sample ( [22], Figure one). Both, PLAGE and the combined z-score are parametric and assume that gene expression profiles are jointly normally distributed. The combined z-score additionally assumes that genes act independently within each gene set. The ssGSEA method from Barbie *et al.*[5] uses the difference in empirical cumulative distribution functions of gene expression ranks inside and outside the gene set to calculate an enrichment statistic per sample which is further normalized by the range of values taken throughout all gene sets and samples.

### Comparison of methods on simulated data

GSVA is unsupervised and yields single sample enrichment scores. Therefore, we can directly compare the performance of GSVA to the combined z-score, single sample GSEA and PLAGE [5,22,35]. However, in contrast to the other methods, GSVA calculates first an expression statistic with the kernel estimation of the ECDF over the samples, which should help in protecting the method against systematic gene specific effects, such as probe effects, and therewith increase its sensitivity. To verify this hypothesis we have performed the following three simulation studies.

**Figure 2:**
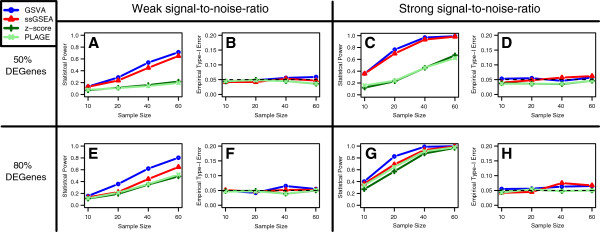
**Comparison of statistical power and type-I error rate between GSVA, PLAGE, single sample GSEA (ssGSEA) and combined z-score (zscore).** The averaged results of 1,000 simulations are depicted as function of the sample size on the *x*-axis, for each of the GSE methods. On the *y*-axis either the statistical power (A, C, E, G) or the empirical type-I error rate (B, D, F, H) is shown. Data were simulated from a linear additive model with sample and probe effects (see Methods) for *p*=1,000 genes. GSE scores were calculated with each method with respect to two gene sets, one of them differentially expressed (DE) and the other one not. Statistical power and empirical type-I error rates were estimated by performing a *t*-test on the DE and non-DE gene sets, respectively, at a significance level of *α*=0.05. These simulations were carried out under the following four different scenarios for the DE gene set: (**A**,**B**) weak signal-to-noise ratio, 50% of DE genes in the DE gene set; (**C**,**D**) strong signal-to-noise ratio, 50% of DE genes in the DE gene set; (**E**, **F**) weak signal-to-noise ratio, 80% of DE genes in the DE gene set; (**G**, **H**) strong signal-to-noise ratio, 80% of DE genes in the DE gene set.

In the first study, we simulated microarray data from a linear additive model with sample and probe effects for *p*=1,000 genes and two groups of samples (see Methods). Using this model we have generated data sets of increasing sample size and defined two gene sets formed by 30 genes each, where one gene set is differentially expressed (DE) and the other is not. For the DE gene set we considered strong and weak signal-to-noise ratios and two different fractions of DE genes (50% and 80%) resulting in four different simulation scenarios. Using the simulated data from each scenario, we have calculated pathway activity profiles with the four sample-wise GSE methods (GSVA, ssGSEA, PLAGE and the combined z-score) and applied a *t*-test on the DE and non-DE gene sets between the two groups of samples. Using the DE gene set and a significance threshold of *α*=0.05, we have estimated the statistical power of each method as function of the sample size. On the same data, but using the non-DE gene set, we have estimated the empirical type-I error rate at *α*=0.05. The results of this simulation in Figure 2 show that GSVA attains higher statistical power than the other three methods in each of the four simulated scenarios while providing similar control of the type-I error rate.

**Figure 3:**
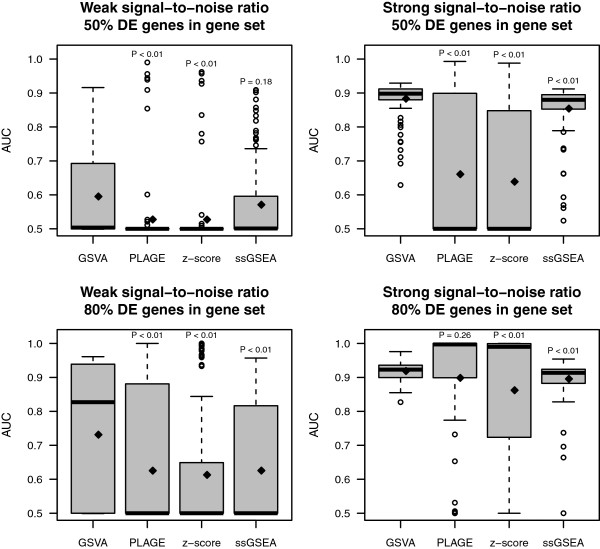
**Comparison of differential pathway activity identification of GSVA, PLAGE, single sample GSEA (ssGSEA) and combined z-score (zscore).** Each panel shows the area under the ROC curve (AUC) on the *y*-axis for differentially expressed genes predicted by each method at 5% FDR over 100 simulations (see Methods). On top of each boxplot the p-value of the *t*-test for no difference in means between GSVA and the corresponding method is reported. The two panels on top correspond to simulations where 50% of the genes in DE gene sets were DE while the two at the bottom contained 80% of DE genes on those DE gene sets. The two panels on the left correspond to a weak signal-to-noise ratio in the DE magnitude while the two on the right correspond to a strong one. Diamonds indicate mean values in boxplots.

In the second simulation study, we compared the accuracy of each GSE method to identify differential pathway activity by calling DE gene sets. For this, we used the previously defined four simulation scenarios as well as the linear additive model with a fixed sample size of *n*=60 and *p*=10,000 genes to simulate data of more realistic dimensions. We set the first 2,000 genes as DE and simulated 1,000 gene sets of which we defined 500 as DE (see Methods). For each simulated gene expression data set, GSE scores were calculated and a two-sample *t*-test was employed to call DE gene sets at 5% FDR. The performance of each GSE method was measured by the area under the ROC curve (AUC) across 100 independent simulations (see Methods). AUC values were calculated from the binary vector of DE calls to compare the ability of each method to identify DE gene sets at a genome-wide significance level. The results are shown in Figure 3. This figure shows that GSVA attains significantly higher mean AUC values than the other GSE methods (*P*<0.05) in all but two of the twelve pairwise comparisons. This improvement in performance of GSVA over the other methods is also observed at a more stringent FDR cutoff of 1% (Additional file 1: Figure S2).

Finally, we carried out a third simulation study in the context of survival analysis. We used again the former linear additive model to simulate microarray data with *p*=1,000 genes and two groups of samples. This time, however, we performed a cross-validation study to assess predictive power using 50 gene sets, each consisting of 10 genes. One of the gene sets was set as DE between the two sample groups while the other 49 were not DE and formed by sampling uniformly at random among the other 990 genes. We used a fixed configuration on the magnitude of differential expression (strong) and on the fraction of DE genes in the DE gene set (50%). In a similar way to the survival simulation by Bair and Tibshirani [36], we generated survival times and censoring status for each observation with different parameters for each group of samples (see Methods). This setting was generated twice to have independent training and test data sets.

GSE scores were calculated separately on the training and test data. A Cox proportional hazards model (Cox PHM) was fitted to each GSE score profile in the training data. The model with the lowest p-value provided by the Wald test was used to predict risk on the test data. As baseline comparison, we also fitted a Cox PHM to each gene expression profile on the training data and selected the 10 genes, corresponding to the gene set size across all gene sets, with lowest p-values given by the Wald test to also predict risk on the test data.

**Figure 4:**
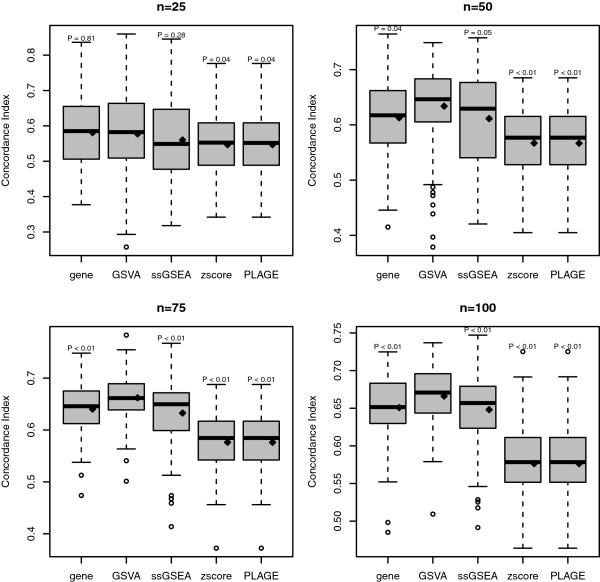
**Comparison of the predictive power for survival analysis of gene-level, GSVA, PLAGE, single sample GSEA (ssGSEA) and combined z-score (zscore) on simulated data.** Each panel corresponds to a different sample size of the simulated data. The *y*-axis shows the concordance index values of predicting survival risk on test data from 100 independent simulations. On top of each boxplot the p-value of the *t*-test for no difference in means between GSVA and the corresponding method is reported. The method *gene* refers to a simple gene-level survival model built from the top 10 genes with lowest p-values reported by the Wald test performed on the training data. Diamonds indicate mean values in boxplots.

The performance of each gene set and gene-level model (using 10 genes) on the test data was assessed by the concordance index. This simulation was repeated 100 times and four entire runs were performed on increasing sample sizes *n*={25,50,75,100} of the simulated data. In Figure 4 the distribution of concordance index values is reported separately for each method and sample size. GSVA provides higher mean and median concordance index values than the other methods at every of the four sample sizes and the difference in means is significant (*P*<0.05) when *n*≥50.

### Lymphoblastic Leukemia: ALL vs MLL

A canonical use of pathway-centric methods is the study of how pathway or gene set variation reveals the underlying biological structure with respect to a given phenotype. An example of this type of analysis was demonstrated in Verhaak, *et al*[28], where they showed how murine-derived neuronal gene sets revealed a corresponding structure for glioblastoma subtypes in a large human cohort. To assess the higher power of GSVA to detect differentially expressed gene sets relevant to a phenotype of interest in real data, we have used a human leukemia data set. The data set consists of 37 different individuals with leukemia, of which 20 correspond to acute lymphoblastic leukemia (ALL) and 17 to mixed-lineage leukemia (MLL) [37]. We assessed the performance of the four sample-wise GSE methods by evaluating their ability to produce a signature of the phenotype ALL vs MLL within different scenarios of magnitude of expression change.

**Figure 5:**
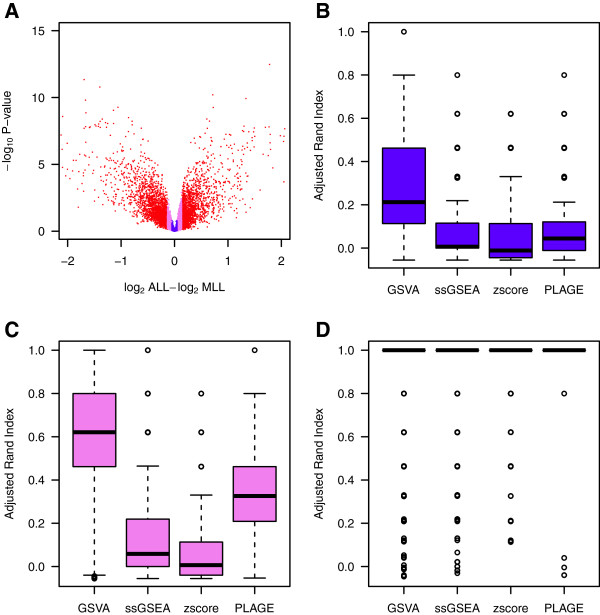
**Comparison of differential pathway activity identification of GSVA, PLAGE, single sample GSEA (ssGSEA) and combined z-score (zscore) on a leukemia data set.** (**A**) Volcano plot of gene expression changes in the Leukemia data set. Genes highlighted in red form the first tercile of largest absolute fold changes, violet indicates the second tercile and blue the third tercile. (**B-D**) Adjusted rand index (ARI) indicating the accuracy of classifying the two groups of samples by hierarchical clustering of the enrichment scores produced by each of the compared methods at the top-5 differentially activated gene sets. The distribution of ARI values is formed by bootstrapping 1,000 times 10 samples from each sample group. Colors match the key given for genes in the volcano plot of (A) and show that, as expected, genes with larger fold changes lead to larger ARI values. However, when fold changes are small (**B-C**) and the underlying signature becomes extremely subtle, GSVA produces enrichment scores that lead to differentially activated gene sets which classify the two sample groups substantially better than using ssGSEA, zscore or PLAGE.

We began by ranking all genes by fold change. Then, we partitioned this ranking into three equally sized fractions depicted in red, violet and blue in the volcano plot in Figure 5, panel A. We used each tercile of genes with increasing fold changes and bootstrap 10 samples from each class 1000 times. We applied the four GSE methods to the bootstrapped data together with the canonical Broad C2 collection of gene sets [4]. Subsequently, we performed differential expression analysis on the enrichment scores using limma [38]. From each ranking of adjusted p-values we selected the top 5 gene sets and used their enrichment scores to make a hierarchical clustering of the samples. We finally partitioned the samples into two groups using the two main branches of the hierarchy and calculated the adjusted rand index (ARI) [39] with respect to the corresponding sample label to assess the robustness of the clustering.

As Figure 5 shows, ARI values depend on the tercile of fold change magnitude considered. Except in the case of the genes belonging to the tercile with largest fold changes (panel D), GSVA produced enrichment scores that led to significantly higher ARI values (*t*-test for difference in means p-value <2*e*−16) than ssGSEA, PLAGE or the combined z-score approaches, demonstrating the larger power of GSVA to produce signatures capable of detecting subtle gene expression changes. Sample-wise enrichment scores easily enable extending this kind of analysis to a more complex phenotype with three or more sample groups. Such an example using adrenocortical carcinoma data can be found in Additional file 1: Figure S3 and Table S1.

### Survival analysis in ovarian carcinoma

**Figure 6:**
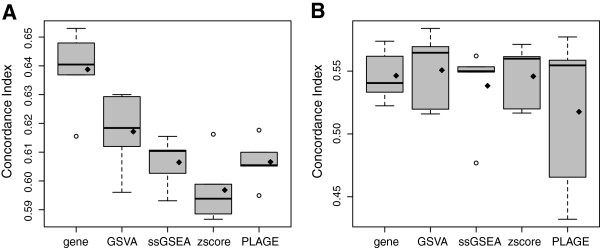
**Survival analysis in a TCGA ovarian cancer data set.** Predictive performance in the survival analysis of a TCGA ovarian cancer microarray data set of *n*=588 samples, measured by the concordance index obtained from a 5-fold cross-validation from (**A**) the training data and (**B**) the test data. Diamonds indicate means in boxplots. Except in the training data using the gene-level model, GSVA provides higher mean and median concordance index values than the other compared methods in both training and testing cross-validated data sets.

We next examined pathway models for predicting patient survival in ovarian serous cystadenocarcinoma (OV). We used a large gene expression experiment (*n*=588) from TCGA [40] to obtain pathway enrichment scores for each of the canonical gene sets (C2) in MSigDB, and compared the four GSE methods. We performed a five-fold cross-validation and calculated GSE scores separately on each training and testing partition of the data with each of the four compared methods. We also considered the original expression data for a simple gene-level model. On each of the training data sets, we fitted a Cox PHM for each gene set, and each gene, in the gene-level model. Then, we selected those five gene-sets, or genes in the gene-level model, with the lowest p-value of the Wald test for no effect on survival. Using the selected gene-sets, we fitted again a Cox PHM on the training data and used it to predict risk on the training and test data sets of GSE scores. We repeated this for the gene-level model. Finally, we assessed the predictive performance of those models, each of them representing a different method, by calculating the concordance index of the predicted risk. As Figure 6 shows, except for the training data set using the gene-level model, GSVA attains higher mean and median concordance index values than the other methods in both, training and testing data sets.

One of the main benefits of pathway-centric approaches is the interpretability they provide in understanding the mechanisms of disease. In Table 1, we list the top gene sets associated to survival as identified by GSVA (a complete list is available in Additional file 1: Table S2). False discovery rates (FDR) are re-estimated using a permutation based approach by randomly ordering the sample labels (patient survival times) 100 times, resulting in FDR estimates of 0.05 and 0.2 for p-value thresholds of 10^−4^ and 5·10^−3^, respectively. The first and second ranked gene sets suggest two important survival mechanisms: DNA repair and modulation of the innate and adaptive immunity, respectively. Further inspection of the top significant gene sets (*P*<10^−3^) show that many of them are involved in wound and immune response. Interestingly, the 3rd and 13th ranked gene sets are derived from response signatures to tretinoin treatment, an all-trans retinoic acid drug that has been shown to suppress growth in ovarian cancer cell lines [41]*,*[42]. Finally, among the top 20 gene sets we note the presence of several EGF and RAS related pathways. While EGFR and RAS mutants are not commonly observed in ovarian cancer [43], activation of these well-studied oncogenes may still play an important role in progression and survival in ovarian cancer.

**Table 1:** Top 5 pathways predictive of survival in ovarian cancer

**Pathways**	**Cox P-value**
SIMBULAN_UV_RESPONSE_NORMAL_DN	7.21×10^−6^
BIOCARTA_VIP_PATHWAY	1.38×10^−5^
ZIRN_TRETINOIN_RESPONSE_WT1_UP	3.38×10^−5^
DASU_IL6_SIGNALING_SCAR_UP	3.46×10^−5^
WANG_HCP_PROSTATE_CANCER	3.65×10^−5^

FDR evaluated as.05 with p-value threshold of 10^−4^.

### GSVA for RNA-seq data

The application of high-throughput sequencing to interrogate RNA concentration in biological samples, popularly known as RNA-seq, is steadily becoming the technology of choice to profile gene expression [44]. The resulting sequence-based measurements take the form of discrete count data and yield a larger dynamic range and unbiased power than microarray technology to survey the cellular state of entire transcriptomes. The nature of these data, however, often requires specific statistical models and bioinformatic methods for their analysis, as in the case of differential expression analysis [45]. This is also the case of many GSE methods developed for microarray data which make distributional assumptions that preclude their direct application to RNA-seq count data [1]*,*[46].

To our knowledge, no attempt has been made to condense gene-level RNA-seq expression profiles into gene sets to capture subtle changes in gene expression. GSE methods exist that either work with closed lists of differentially expressed genes (e.g. topGO [47], GOseq [48]), or rankings of some differential expression statistic, such as GSEA [4] and the mean-rank gene set enrichment method [49]. GOseq [48] is specifically designed to address gene length biases in lists of differentially expressed genes derived from RNA-seq data. But GOseq ignores genes that are not considered as differentially expressed and removes them from the analysis, hence ignoring genes with subtle changes. Also, rank-based methods ignore relative changes of genes in a pathway resulting in equal treatment of the genes, although they might have different fold changes [50]. Hence, these methods may be underpowered to detect subtle changes in pathway activity.

**Figure 7:**
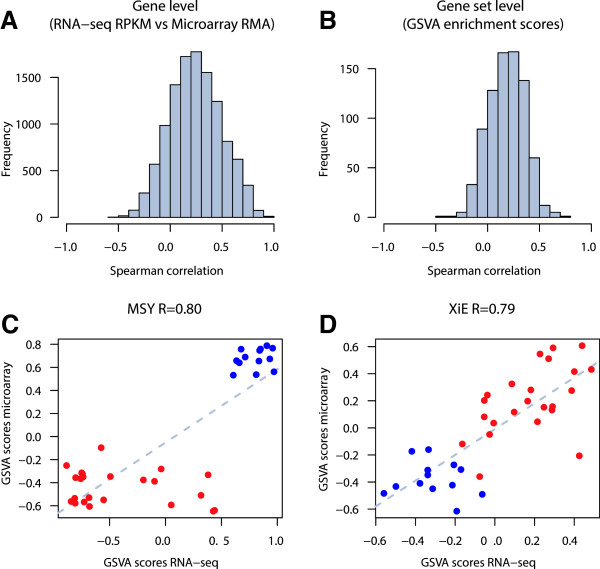
**GSVA for RNA-seq (Argonne). A.** Distribution of Spearman correlation values between gene expression profiles of RNA-seq and microarray data. **B.** Distribution of Spearman correlation values between GSVA enrichment scores of gene sets calculated from RNA-seq and microarray data. **C** and **D.** Comparison of GSVA enrichment scores obtained from microarray and RNA-seq data for two gene sets containing genes with sex-specific expression: MSY formed by genes of the male-specific region of the Y chromosome (male-specific), and XiE formed by genes that escape X-inactivation in females (female-specific). Red and blue points represent female and male samples, respectively. In both cases GSVA scores show very high correlation between the two profiling technologies where female samples show higher enrichment scores in the female-specific gene set and male samples show higher enrichment scores in the male-specific gene set.

Here, we show how to apply GSVA to RNA-seq data. We provide pathway activity profiles analogous to the ones obtained from microarray data by using samples of lymphoblastoid cell lines (LCL) from HapMap individuals which have been profiled using both technologies [51]*,*[52]. Microarray and RNA-seq data were processed to obtain gene expression data matrices with matching gene and sample identifiers (Methods). The RNA-seq data consists of two tables of counts derived from reads obtained at two different sequencing centers, denoted by Argonne and Yale; see [52]. We calculated Spearman correlations for all genes and gene sets from both technologies. The resulting distributions of correlation values are shown in Figure 7, panels A and B, using the Argonne RNA-seq data (see Additional file 1: Figure S4 for analogous results for the Yale RNA-seq data). We show that GSVA enrichment scores correlate similarly to gene expression levels produced by both profiling technologies.

We also examined two gene sets containing gender-specific genes in detail: genes that escape X-inactivation in female samples [53] and genes that are located on the male-specific region of the Y chrosomome [54]. Figure 7 illustrates that microarray and RNA-seq enrichment scores correlate very well in these gene sets, with *ρ*=0.82 for the male-specific gene set and *ρ*=0.78 for the female-specific gene set. Male and female samples show higher GSVA enrichment scores in their corresponding gene sets. This demonstrates the flexibility of GSVA to enable analogous unsupervised and single sample GSE analyses in data coming from both microarray and RNA-seq technologies.

## Methods

### Simulations

The simulation studies were carried out using the following linear additive model for mimicking normalized microarray data on *p* genes and *n* samples divided in two groups representing a case-control scenario:

(6) $$y_{\text{ij}}=\alpha_{i}+\beta_{j}+\in_{\text{ij}}\;,$$

where $\alpha_{i}∼N(\mu =0,\sigma =1)$ is a gene-specific effect, such as a probe-effect, with i=1,…,p, $\beta_{j}∼N(\mu_{j},\sigma_{j})$ is a sample-effect with *j*=1,2 and $e_{\text{ij}}∼N(\mu =0,\sigma =1)$ corresponds to random noise.

When assessing statistical power and type-I error in Figure 2, we set *p*=1,000 genes, out of which the first 30 were considered to form a DE gene set and the next 30 a non-DE gene set. We considered four different sample sizes *n*={10,20,40,60} and two varying conditions leading to four different simulation scenarios: the fraction of differentially expressed genes in the gene set (50% and 80%) and the signal-to-noise ratio expressed as the magnitude of the mean sample effect in DE genes for one of the sample groups (weak and strong signal-to-noise ratio). For non-DE genes *μ*_1_=*μ*_2_=0 with *σ*_1_=*σ*_2_=1 while for DE genes *μ*_2_=0.5 for the weak effect, *μ*_2_=1 for the strong effect and *σ*_2_=0.5. Using the model in Eq. (6) with these parameters, we simulated 1,000 independent data sets. For each of the four GSE methods we obtained a GSE score matrix for two gene sets (DE and non-DE) by *n* samples. On each GSE score matrix, we performed a two-sample *t*-test on the two gene sets for a difference in mean between the two groups of samples (*H*_0_:*μ*_1_−*μ*_2_=0) at a significance level *α*=0.05. The statistical power was then estimated as 1 minus the fraction of non-rejections of the DE gene set and the empirical type-I error was estimated as the fraction of rejections of the non-DE gene set, across the 1,000 simulations.

In the second simulation study we considered *p*=10,000 genes out of which 2,000 were set as DE and from which 1,000 gene sets were built, 500 of them being DE. DE genes and gene sets were simulated using the previously described parameters and simulation scenarios. Non-DE gene sets were simulated by sampling from the *p*=10,000 genes uniformly at random while DE gene sets were simulated by sampling among DE and non-DE genes in the proportions (50% or 80% of DE genes) defined by the corresponding scenario. For each scenario, we sampled the data this way 100 times and calculated GSE scores using the four GSE methods for every resulting data set. Using those GSE scores we performed a two-sample *t*-test for each gene set and called DE those meeting FDR cutoffs of 5% and 1%. Performance was assessed by calculating ROC curves and AUC values using the R package ROCR[55].

The simulation study assessing the predictive power of GSE scores for survival in Figure 4 was performed using linear additive model in Eq. 6, where *μ*_2_=1 was fixed for DE genes in one of the sample groups. Survival times were generated for each sample group from two normal distributions N(μ=6,σ=2) and N(μ=10,σ=2). Censoring times were generated from a normal distribution N(μ=10,σ=3). A sample was considered to be censored when the censoring time was smaller than the survival time.

### Data

Data for differential expression analysis was obtained from the following sources: Leukemia [37] (http://www.broadinstitute.org) and Adrenocortical Carcinoma [56] (http://www.ncbi.nlm.nih.gov/geo; GSE10927). Data for the ovarian analysis was downloaded from TCGA on April 2011. At the time of analysis, 389 samples were available that had clinical data, gene expression (Affy U133A), and CNV (Affy SNP 6.0). In all cases, TCGA Level 3 data was used. Gene expression data was batch corrected using ComBat [57]. RNA-seq data corresponded to HapMap [58] lymphoblastoid cell lines (LCL) of Yoruba individuals [52] and the processed tables of counts were downloaded from http://eqtl.uchicago.edu/RNA_Seq_data/results. Matching microarray samples form part of a larger study by Huang and co-workers [51] (http://www.ncbi.nlm.nih.gov/geo; GSE7792).

### Microarray data processing

Data analysis was performed using the R [59] and Bioconductor [60] software. We selected chips which passed quality control using affyPLM [61]. AffyPLM fits models on probe set level to identify chips of lower quality. Relative Log Expression (RLE) values (comparing probe expression on each array against the median expression across all arrays) and Normalized Unscaled Standard Errors (NUSE) (standard error estimates obtained for each gene and standardized across arrays) are calculated and cut-offs applied to remove low-quality samples.

Chips whose processing batch was confounded with the outcome of interest are not considered in the analysis. Each remaining Affymetrix chip was background adjusted, normalized and log2 transformed using the Robust Multi-array Average (RMA) algorithm [62].

Genes that are not expressed over the detection level of the microarray or whose expression values have a limited variability through the samples do not provide discriminatory power and may compromise the statistical power of subsequent analyses. For this reason, we removed 50% of the genes with lower variability as measured by the interquartile range (IQR) across the samples except in the LCL microarray data.

### RNA-seq data processing

The RNA-seq data from Pickrell *et al.* (2010) [52] were produced at two sequencing centers, Argonne and Yale, and preprocessed by the authors into two separate tables of counts of 41,466 Ensembl genes by 80 and 81 samples, respectively. We use these two tables of counts, and refer the reader for details on read mapping and summarization into gene-level counts to the methods of the publication [52]. Some of the samples (11 from Argonne and 12 from Yale) were prepared and sequenced twice within each sequencing center. In these cases we kept the sample of deeper coverage obtaining a final number of 69 samples on each table. We further filtered genes with low expression by discarding those with a mean of less than 0.5 counts per million calculated in log2 scale resulting in tables of counts with 17,607 genes (Argonne) and 17,843 genes (Yale) by 69 samples and we kept genes present in both tables (17,324). Next, we normalized these two tables of counts adjusting for gene length and G+C content using the Bioconductor package cqn [24]. The corresponding gene length and G+C content information was extracted from data deposited at the same site from where the tables of counts were downloaded.

In order to proceed with the comparison of GSVA enrichment scores between microarray and RNA-seq data, we further filtered these two normalized tables of counts in order to match the genes and samples obtained after processing the LCL microarray data from Huang and co-workers [51]. This step required first to translate Ensembl gene identifiers into Entrez gene identifiers and second to match gene and sample identifiers between microarray and RNA-seq data. After these two steps we obtained the two final tables of counts analyzed in this paper of 11,508 Entrez genes by 36 samples from which 23 correspond to female and 13 to male individuals.

### Gene sets database

In all experiments, we used the gene sets database from the Molecular Signature Database version 3 (MSigDB) C2 collection (curated pathways) [4] with 833 canonical pathways and 2392 chemical and gene perturbations, unless otherwise stated. After mapping genes from an experiment to the gene set database, we ignore all gene sets with fewer than 10 genes or more than 500 genes.

### FDR and multiple hypothesis correction

In most experiments, we use a permutation approach to estimate an empirical FDR at a specified p-value threshold. In several cases we report multiple hypothesis correction based on the Benjamini-Hochberg (B.H.) approach [63] to obtain corrected p-values. In general, multiple hypothesis correction on gene sets is problematic, as many gene sets are highly overlapping and therefore not merely correlated, but essentially duplicated. Our use of B.H. is likely a conservative estimate of FDR and therefore used primarily as a demonstration of statistical power.

## Discussion

The analyses conducted on simulated and real data demonstrate that GSVA outperforms competing methods for modeling pathway variation across samples in the context of identification of differential pathway activity and survival analysis. However, given the large number of GSE methods published and available to the bioinformatic community, GSVA may not be the optimal tool for every expression data set. We recommend GSVA as an intermediate universal tool, providing summaries of pathway activity for more open-ended biological analysis. For specific applications, highly specialized algorithms optimized for addressing domain specific problems may outperform GSVA. The user should also be aware that the non-parametric density estimation within the GSVA algorithm requires a sufficient number of observations which, according to our analysis of statistical power in Figure 2, should be larger than *n*=10.

Non-specific filtering of genes in high-throughput experiments has been shown to increase the statistical power to detect significant changes in gene expression levels [64] and this observation is likely to hold at gene set level. We have used a simple non-specific filtering strategy of a minimum and maximum cutoff on the size of a gene set after gene identifiers have been matched between gene expression data and gene sets. However, other strategies based on expected features of biologically relevant gene sets could potentially be more helpful. For instance, genes that are part of the same gene set or pathway are more likely to be expressed coordinately and are expected to exhibit some degree of correlation. Gene sets containing correlated genes are more coherent and provide a higher biological signal than incoherent, uncorrelated gene sets [65]. Hence, removing functionally incoherent pathways could constitute an appealing non-specific filtering strategy to improve detection power at gene set level.

## Conclusions

We have presented a method for assaying the variation of gene set enrichment over a sample population. The method is freely available as a Bioconductor package for R under the name GSVA at http://www.bioconductor.org. The increasing availability of large data sets with multiple assays and complex phenotypes has motivated our work because the study of these data sets within the context of pathways will be critical to their understanding. The GSVA method is both non-parametric and unsupervised, and bypasses the conventional approach of explicitly modeling phenotypes within the enrichment scoring algorithm. We have also shown how GSVA can be easily adapted to the analysis of RNA-seq data producing results analogous to its microarray counterpart. In the Additional file 1, two other examples of GSVA applications can be found including differential pathway analysis in a multi-class adrenocortical carcinoma data set (Additional file 1: Figure S3 and Table S1), and correlation analysis of pathways and copy-number alterations in ovarian carcinoma (Additional file 1: Figure S5).

For future directions, we believe GSVA may be used in genetical genomics strategies analogous to eQTL mapping to study, what we might call, pathway-QTL to identify DNA polymorphisms that impact pathway activity [66]. This could be extended further to support causal inference [67], where pathways replace genes in modeling the causal chain of genotype → gene expression → phenotype.

## Availability and requirements

•**Project name:** GSVA

•**Project home page:**http://www.bioconductor.org/packages/release/bioc/html/GSVA.html

•**Operating system(s):** Platform independent

•**Programming language:** R, C

•**Other requirements:** R (>= 2.15.0), the R package methods, and the Bioconductor package GSEABase (>= 1.18.0)

•**License:** GPL (>= 2)

•**Any restrictions to use by non-academics:** no restrictions

## Competing interests

The authors declare no conflict of interest.

## Authors’ contributions

JG conceived and designed the GSVA algorithm. JG and RC implemented the software. SH and JG conceived and designed the applications of GSVA. SH, RC and JG analyzed the data and wrote the paper. All authors read and approved the final manuscript.

## Supplementary Material

Additional file 1 Supplementary material including figures S1 to S5 and tables S1 and S2. Click here for file
