# Supplementary material for: GSVA: gene set variation analysis for microarray and RNA-Seq data
Source: BMC Bioinformatics. 2013 Jan 16;14:7. doi: 10.1186/1471-2105-14-7 (PMC3618321; doi:10.1186/1471-2105-14-7)
Supplement: Additional file 1 — Supplementary material including figures S1 to S5 and tables S1 and S2. [file 1471-2105-14-7-S1.pdf]

SUPPLEMENTARY MATERIAL

GSVA: Gene Set Variation Analysis

Sonja Hänzelmann
shanzelmann@imim.es

Research Program on Biomedical Informatics (GRIB), Hospital del Mar Medical Research Institute (IMIM) and Universitat Pompeu Fabra, Barcelona, Catalonia, Spain

Robert Castelo[1]
robert.castelo@upf.edu

Research Program on Biomedical Informatics (GRIB), Hospital del Mar Medical Research Institute (IMIM) and Universitat Pompeu Fabra, Barcelona, Catalonia, Spain

and

Justin Guinney[1]
justin.guinney@sagebase.org

Sage Bionetworks, 1100 Fairview Ave N., Seattle, Washington, 98109 USA

November 30, 2012

[1]To whom correspondence should be addressed.

1 Distribution of GSVA scores

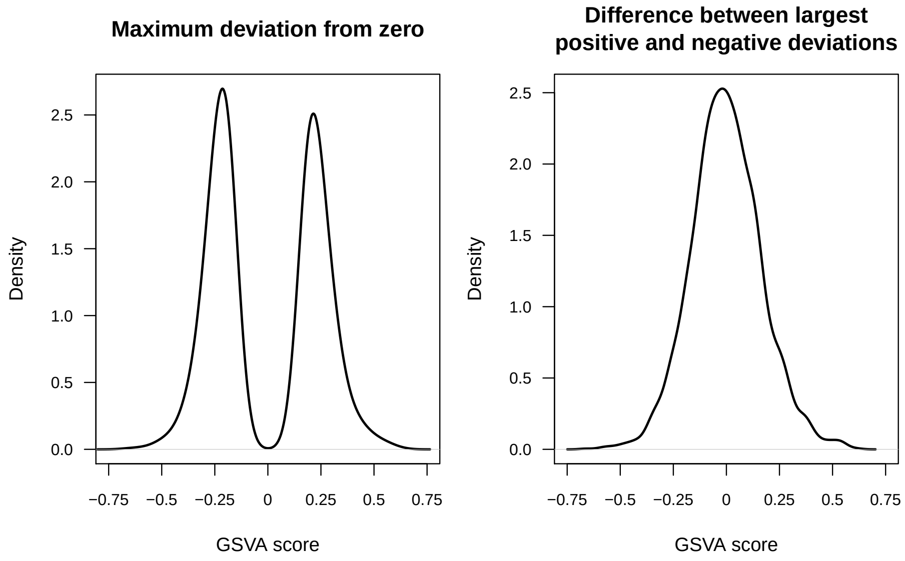

Figure S1: Distribution of GSVA scores. The left plot shows the distribution of GSVA scores calculated from standard Gaussian deviates $\mathcal{N}(\mu = 0, \sigma = 1)$ on $p = 20,000$ genes and $n = 30$ samples using 100 gene sets with sizes uniformly sampled from 10 to 100 genes using the classical maximum deviation enrichment score from a Kolmogorov-Smirnov random walk. The right plot shows normalized enrichment scores (see main paper) calculated from the same simulated data.

2 Accuracy of single-sample GSE methods by simulation

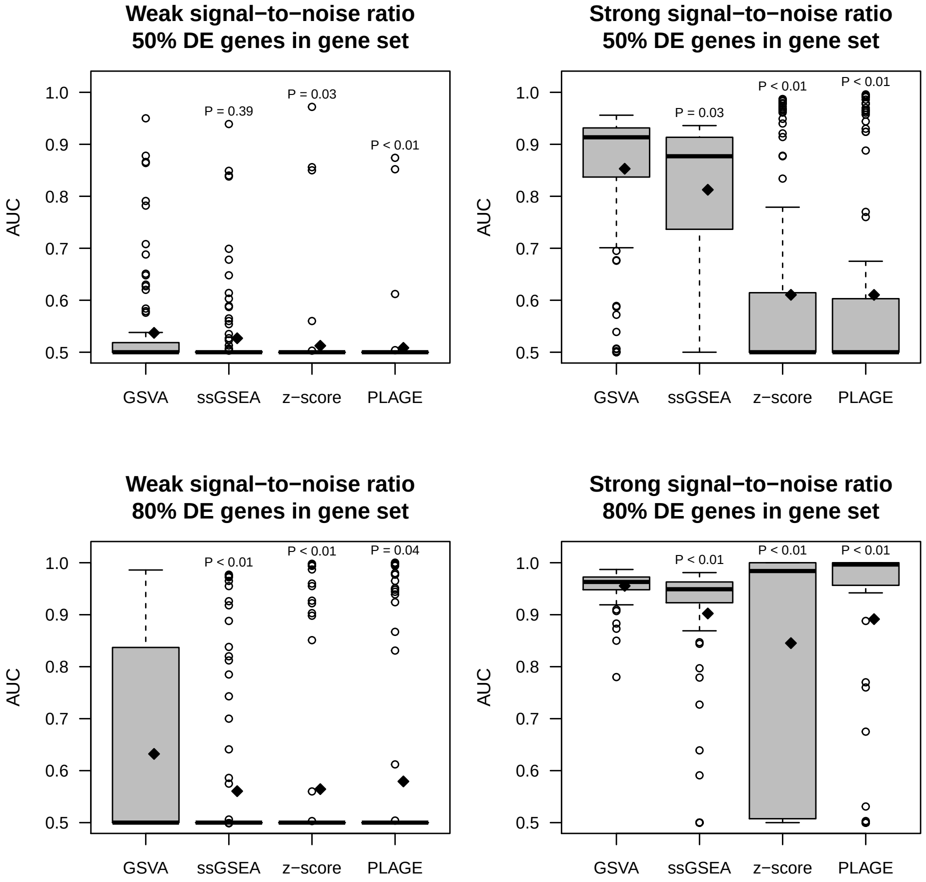

Figure S2: **Comparison of differential expression prediction of GSVA, PLAGE, single sample GSEA (ssGSEA) and combined z-score (zscore).** Each panel shows the area under the ROC curve (AUC) in the y-axis for differentially expressed genes predicted by each method at 1% FDR on 100 simulations (see Methods). The two panels on top correspond to simulations where 50% of the genes in DE gene sets were DE while the two at the bottom contained 80% of DE genes on those DE gene sets. The two panels on the left correspond to a weak signal-to-noise ratio in the DE magnitude while the two on the right correspond to a strong one. Diamonds indicate mean values in boxplots.

3 Identification of significant pathways

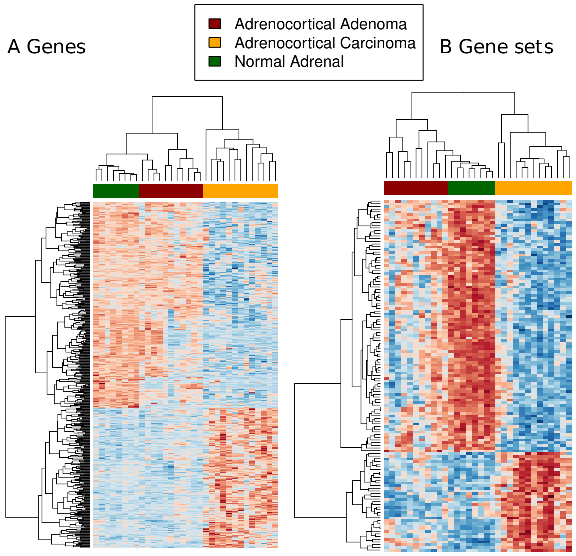

Figure S3: Differential expression analysis of adrenocortical carcinoma. Heatmaps of genes expression values (A) and GSVA pathway scores (B) for genes and pathways that change significantly across the three subtypes.

The transformation of gene expression levels into GSVA enrichment scores permits gene set enrichment analyses as a classical differential gene expression analysis. To identify gene sets that are differentially activated between the sample groups, we use linear models and moderated t-statistics. These are computed using the empirical Bayes shrinkage method implemented in the Bioconductor package limma [1]. The combination of utilizing GSVA scores and limma allows for the assessment of gene set enrichment directly in complex experimental designs including multiple sample groups and batch effects. We illustrate this application by means of an adrenocortical carcinoma data set with the three groups, adrenocortical carcinoma (ACC), adenoma (ACA) and normal adrenal tissue (NAC) [2].

Adenoma is a benign form of adrenocortical tumors and found in the majority of patients, whereas the malignant tumor (adrenocarcinoma) has an estimated annual incidence of one to two per million population [3]. Most genes disrupted in adrenocarcinoma are either tumor suppressors or oncogenes, such as insulin-like growth factor 1 (IGF1), β-catenin, steroid factor 1 (SF1) or growth factors (details in Table S1 below). ACA and NA tissues exhibit a very similar molecular profile, which leads to difficulties in illuminating the subtle underlying changes. Hence, we apply a non-specific gene and gene set filter, low variation genes and small gene sets are removed, before executing the GSVA function. This approach helps to focus on gene sets representing a more coherent struc-

ture and emphasize the fine differences between the three groups. We model the pairwise contrasts of ACA, ACC and normal tissue to identify genes/gene sets that are differentially expressed and can be utilized for clustering. For this, we use a moderated t-statistic [1] and take the intersection of significant gene sets (at a corrected p-value of .001). Subsequently, we use unsupervised clustering on the selected gene sets to generate the heatmap in Figure S3B. Similarly, we perform a gene-centric analysis with the results showing in Figure S3A. In both cases, the use of pathways or genes is capable of separating the three groups, demonstrating GSVA enrichment scores may be used in an analogous fashion to gene expression for modeling multiple groups. The added value of GSVA is that pathways/gene sets illuminate the subtle differences between adenoma and normal tissue clearer.

Table S1: Gene sets showing different alteration patterns in adrenocortical carcinoma (ACC), adrenocortical adenoma (ACA) and normal adrenal cortices (NA).

**Gene sets significantly altered (FDR 10^{-5})**	**Gene sets distinguishing adrenocortical carcinoma (ACC), adrenocortical adenoma (ACA) and normal adrenal cortices (NA)**
SABATES COLORECTAL ADENOMA DN	*Wnt* signaling target genes are contained in this data set, which are frequently activated in ACC [4].
HATADA METHYLATED IN LUNG CANCER UP; SMID BREAST CANCER BASAL DN; POOLA INVASIVE BREAST CANCER DN; PYEON CANCER HEAD AND NECK VS CERVICAL UP; GEORGES TARGETS OF MIR AND MIR; LIU PROSTATE CANCER DN; CASORELLI ACUTE PROMYELOCYTIC LEUKEMIA DN; DAZARD UV RESPONSE CLUSTER G;	These gene sets contain growth factors (*IGF1* (Insulin Growth Factor 1), *VEGF* (Vascular Endothelial Growth Factor)), estrogen receptor (*ER*) and its targets, as well as *TP53* (Cellular tumor antigen p53) and genes involved in cell cycle regulation (cyclins, cell division kinases). All of these genes are associated with ACC [5]. Early stage ACC correlates with *ER* expression [6]. Topoisomerase II α has been shown to distinguish adenomas from carcinomas in ACC [7]. *BUB1B* (also contained in the gene sets on the left) has been identified as a marker for survival [8].
ODONNELL TFRC TARGETS UP; GEORGES TARGETS OF MIR AND MIR; CHEOK RESPONSE TO MERCAPTOPURINE UP; KEGG P SIGNALING PATHWAY; BIOCARTA G PATHWAY; BIOCARTA P PATHWAY; BIOCARTA ATRBRCA PATHWAY	The overexpression of gene sets involved in cell cycle regulation (cyclins, cell division kinases) and *TP53* agrees with findings from previous functional studies in ACC [2], [5]. Also, p53 tumor suppressor pathways lead to changes from normal cells to tumor cells [9]
DELYS THYROID CANCER DN	Both retinoic acid production and aldehyde dehydrogenases were found to be decreased in ACC [8],[5].

4 Survival analysis in ovarian serous carcinoma

In the main paper we describe how pathways can be used to predict survival in ovarian cancer. We fit a Cox proportional hazards model to each gene set to identify predictive pathways, using a permutation approach to estimate FDR. Below we list the most predictive pathways (FDR < .1).

Table S2: Top ranking ovarian (OV) cancer pathways, predictive of survival.

SIMBULAN UV RESPONSE NORMAL DN	7.21E-06
BIOCARTA VIP PATHWAY	1.39E-05
ZIRN TRETINOIN RESPONSE WT1 UP	3.38E-05
DASU IL6 SIGNALING SCAR UP	3.46E-05
WANG HCP PROSTATE CANCER	3.65E-05
CHASSOT SKIN WOUND	7.04E-05
AMIT EGF RESPONSE 40 HELA	7.97E-05
AMIT EGF RESPONSE 40 MCF10A	8.62E-05
AMIT DELAYED EARLY GENES	1.05E-04
BROWNE HCMV INFECTION 10HR DN	1.07E-04
NAGASHIMA EGF SIGNALING UP	1.07E-04
VANHARANTA UTERINE FIBROID DN	1.32E-04
ZIRN TRETINOIN RESPONSE UP	1.46E-04
YAGI AML WITH INV 16 TRANSLOCATION	1.67E-04
BROWNE HCMV INFECTION 14HR DN	1.79E-04
DANG MYC TARGETS DN	2.09E-04
REACTOME CREB PHOPHORYLATION THROUGH THE ACTIVATION OF RAS	2.63E-04
BREUHAHN GROWTH FACTOR SIGNALING IN LIVER CANCER	2.64E-04
BEGUM TARGETS OF PAX3 FOXO1 FUSION DN	2.74E-04
DAUER STAT3 TARGETS UP	2.88E-04
MOROSETTI FACIOSCAPULOHUMERAL MUSCULAR DISTROPHY DN	2.97E-04
NAGASHIMA NRG1 SIGNALING UP	3.01E-04
CHIARADONNA NEOPLASTIC TRANSFORMATION KRAS DN	3.11E-04
PROVENZANI METASTASIS DN	3.37E-04
BIOCARTA CARDIACEGF PATHWAY	4.32E-04
ICHIBA GRAFT VERSUS HOST DISEASE 35D DN	4.36E-04
CHEN LVAD SUPPORT OF FAILING HEART UP	4.39E-04
GRASEMANN RETINOBLASTOMA WITH 6P AMPLIFICATION	4.58E-04
AMIT EGF RESPONSE 20 HELA	4.61E-04

Table S2 – Continued on next page

Table S2 –continued from previous page

BIOCARTA ETS PATHWAY	4.73E-04
BIOCARTA GSK3 PATHWAY	4.74E-04
KEGG LONG TERM POTENTIATION	4.85E-04
CROONQUIST STROMAL STIMULATION UP	5.41E-04
TURASHVILI BREAST DUCTAL CARCINOMA VS DUCTAL NORMAL DN	5.41E-04
TOMLINS PROSTATE CANCER DN	5.47E-04
BREDEMEYER RAG SIGNALING VIA ATM NOT VIA NFKB UP	5.50E-04
BIOCARTA CALCINEURIN PATHWAY	5.56E-04
ZHU CMV ALL DN	5.65E-04
MCCLUNG DELTA FOSB TARGETS 2WK	5.68E-04
AMIT SERUM RESPONSE 40 MCF10A	6.08E-04
AMIT SERUM RESPONSE 60 MCF10A	6.33E-04
ZHU CMV 8 HR DN	6.40E-04
HOOI ST7 TARGETS UP	6.59E-04
KAYO CALORIE RESTRICTION MUSCLE UP	6.93E-04
BIOCARTA NGF PATHWAY	7.33E-04
DAZARD RESPONSE TO UV NHEK UP	7.58E-04
RODRIGUES THYROID CARCINOMA DN	7.65E-04
AMIT EGF RESPONSE 60 HELA	7.71E-04
ZHU CMV 24 HR DN	8.52E-04
DASU IL6 SIGNALING SCAR DN	8.87E-04
DAZARD UV RESPONSE CLUSTER G24	1.00E-03
SASSON RESPONSE TO GONADOTROPHINS DN	1.02E-03
DACOSTA UV RESPONSE VIA ERCC3 XPCS DN	1.03E-03
KEGG COLORECTAL CANCER	1.05E-03
KIM WT1 TARGETS UP	1.06E-03
IZADPANAH STEM CELL ADIPOSE VS BONE DN	1.06E-03
LEE LIVER CANCER MYC TGFA UP	1.11E-03
TIAN TNF SIGNALING NOT VIA NFKB	1.12E-03
UZONYI RESPONSE TO LEUKOTRIENE AND THROMBIN	1.14E-03
GINESTIER BREAST CANCER 20Q13 AMPLIFICATION UP	1.16E-03
YAO HOXA10 TARGETS VIA PROGESTERONE UP	1.16E-03
SMID BREAST CANCER RELAPSE IN LIVER DN	1.23E-03
BOYLAN MULTIPLE MYELOMA C UP	1.23E-03
LIN SILENCED BY TUMOR MICROENVIRONMENT	1.24E-03
BIOCARTA FCER1 PATHWAY	1.27E-03
RIGGI EWING SARCOMA PROGENITOR DN	1.28E-03
CORRE MULTIPLE MYELOMA DN	1.29E-03
BASSO HAIRY CELL LEUKEMIA DN	1.32E-03
BROWNE HCMV INFECTION 8HR DN	1.33E-03
HALMOS CEBPA TARGETS DN	1.34E-03
VANTVEER BREAST CANCER BRCA1 DN	1.35E-03

Table S2 – Continued on next page

Table S2 –continued from previous page

WHITESIDE CISPLATIN RESISTANCE DN	1.37E-03
RUGO STRESS RESPONSE SUBSET G	1.39E-03
KYNG DNA DAMAGE BY 4NQO OR UV	1.39E-03
CHANG POU5F1 TARGETS UP	1.40E-03
ST GRANULE CELL SURVIVAL PATHWAY	1.44E-03
AMIT EGF RESPONSE 240 HELA	1.45E-03
ABRAHAM ALPC VS MULTIPLE MYELOMA UP	1.46E-03
GERY CEBP TARGETS	1.46E-03
BIOCARTA NFAT PATHWAY	1.47E-03
AMIT EGF RESPONSE 60 MCF10A	1.54E-03
WILLIAMS ESR2 TARGETS UP	1.55E-03
REACTOME SMOOTH MUSCLE CONTRACTION	1.57E-03
SANA TNF SIGNALING DN	1.59E-03
REACTOME POST NMDA RECEPTOR ACTIVATION EVENTS	1.59E-03
BIOCARTA PGC1A PATHWAY	1.66E-03
NIKOLSKY MUTATED AND AMPLIFIED IN BREAST CANCER	1.68E-03
LEE LIVER CANCER CIPROFIBRATE UP	1.74E-03
BONOME OVARIAN CANCER SURVIVAL SUBOPTIMAL DEBULKING	1.76E-03
SIMBULAN UV RESPONSE IMMORTALIZED DN	1.82E-03
BIOCARTA BCR PATHWAY	1.87E-03
SASSON RESPONSE TO FORSKOLIN DN	1.97E-03
KIM WT1 TARGETS 8HR UP	1.97E-03
REACTOME DOWN STREAM SIGNAL TRANSDUCTION	2.05E-03
BERENJENO TRANSFORMED BY RHOA FOREVER DN	2.06E-03
BIOCARTA IGF1 PATHWAY	2.07E-03
VALK AML CLUSTER 10	2.10E-03
BROWNE HCMV INFECTION 20HR DN	2.10E-03
BIOCARTA GPCR PATHWAY	2.13E-03
FRASOR RESPONSE TO ESTRADIOL UP	2.15E-03
DORSAM HOXA9 TARGETS DN	2.17E-03
SMID BREAST CANCER RELAPSE IN PLEURA DN	2.18E-03
MATTIOLI MULTIPLE MYELOMA WITH 14Q32 TRANSLOCATIONS	2.21E-03
KEGG FOCAL ADHESION	2.33E-03
REACTOME THROMBOXANE SIGNALLING THROUGH TP RECEPTOR	2.39E-03
GESERICK TERT TARGETS DN	2.45E-03
VARELA ZMPSTE24 TARGETS UP	2.46E-03
HOFFMANN PRE BI TO LARGE PRE BII LYMPHOCYTE UP	2.48E-03
VALK AML CLUSTER 15	2.51E-03
LEE LIVER CANCER MYC UP	2.54E-03

Table S2 – Continued on next page

Table S2 –continued from previous page

BIOCARTA NDKDYNAMIN PATHWAY	2.60E-03
KUUSELO PANCREATIC CANCER 19Q13 AMPLIFICATION	2.63E-03
LANDIS ERBB2 BREAST TUMORS 324 DN	2.80E-03
CHIARADONNA NEOPLASTIC TRANSFORMATION KRAS CDC25 DN	2.84E-03
FRIDMAN SENESCENCE DN	2.85E-03
LANDIS BREAST CANCER PROGRESSION DN	2.86E-03
BIOCARTA MEF2D PATHWAY	2.88E-03
GOLUB ALL VS AML UP	2.94E-03
KYNG DNA DAMAGE DN	2.95E-03
BROWNE HCMV INFECTION 12HR DN	2.95E-03
ENK UV RESPONSE EPIDERMIS DN	3.03E-03
VERRECCHIA RESPONSE TO TGFB1 C1	3.04E-03
DORN ADENOVIRUS INFECTION 24HR DN	3.08E-03
OUYANG PROSTATE CANCER PROGRESSION UP	3.10E-03
MARTINEZ RESPONSE TO TRABECTEDIN	3.27E-03
REACTOME SIGNALING BY PDGF	3.35E-03
WEST ADRENOCORTICAL TUMOR MARKERS DN	3.36E-03
BIOCARTA PDGF PATHWAY	3.41E-03
LIN MELANOMA COPY NUMBER DN	3.43E-03
LOPEZ MESOTHELIOMA SURVIVAL DN	3.76E-03
DORN ADENOVIRUS INFECTION 48HR DN	3.84E-03
REACTOME OPIOID SIGNALLING	3.85E-03
BROWNE HCMV INFECTION 30MIN UP	3.87E-03
DACOSTA UV RESPONSE VIA ERCC3 TTD DN	3.90E-03
BERENJENO TRANSFORMED BY RHOA DN	3.93E-03
SENESE HDAC1 AND HDAC2 TARGETS DN	4.06E-03
HINATA NFKB TARGETS KERATINOCYTE DN	4.11E-03
KEGG REGULATION OF ACTIN CYTOSKELETON	4.12E-03
GRADE COLON CANCER DN	4.16E-03
MILI PSEUDOPODIA CHEMOTAXIS UP	4.19E-03
TURASHVILI BREAST DUCTAL CARCINOMA VS LOBULAR NORMAL DN	4.23E-03
BERENJENO TRANSFORMED BY RHOA REVERSIBLY DN	4.35E-03
KEGG MAPK SIGNALING PATHWAY	4.39E-03
STEIN ESTROGEN RESPONSE NOT VIA ESRRA	4.43E-03
BILD SRC ONCOGENIC SIGNATURE	4.47E-03
BIOCARTA INTEGRIN PATHWAY	4.57E-03
MILI PSEUDOPODIA	4.60E-03
SHI SPARC TARGETS UP	4.66E-03
NING CHRONIC OBSTRUCTIVE PULMONARY DISEASE DN	4.86E-03
REACTOME SEMA4D IN SEMAPHORIN SIGNALING	4.89E-03
WEST ADRENOCORTICAL TUMOR DN	4.95E-03

Table S2 – Continued on next page

Table S2 –continued from previous page

REACTOME G BETA GAMMA SIGNALLING THROUGH PI3KGAMMA	4.97E-03
KERLEY RESPONSE TO CISPLATIN UP	5.04E-03
BIOCARTA INSULIN PATHWAY	5.07E-03
SU THYMUS	5.20E-03
GENTILE UV RESPONSE CLUSTER D6	5.29E-03
BIOCARTA AT1R PATHWAY	5.29E-03
VERRECCHIA RESPONSE TO TGFB1 C4	5.29E-03
BIOCARTA TPO PATHWAY	5.30E-03
ONDER CDH1 SIGNALING VIA CTNNB1	5.31E-03
HASLINGER B CLL WITH 13Q14 DELETION	5.41E-03
ENGELMANN CANCER PROGENITORS DN	5.44E-03
VALK AML CLUSTER 9	5.53E-03
BERNARD PPAPDC1B TARGETS DN	5.54E-03
AMUNDSON POOR SURVIVAL AFTER GAMMA RADIATION 2G	5.64E-03
WATTEL AUTONOMOUS THYROID ADENOMA DN	5.66E-03
REACTOME NEURORANSMITTER RECEPTOR BINDING AND DOWNSTREAM TRANSMISSION IN THE POSTSYNAPTIC CELL	5.75E-03
SWEET LUNG CANCER KRAS DN	5.78E-03
BENPORATH ES 2	5.86E-03
DORN ADENOVIRUS INFECTION 32HR DN	5.98E-03
MASRI RESISTANCE TO TAMOXIFEN AND AROMATASE INHIBITORS DN	6.02E-03
WU ALZHEIMER DISEASE DN	6.02E-03
MAHAJAN RESPONSE TO IL1A DN	6.07E-03
PETRETTO CARDIAC HYPERTROPHY	6.08E-03
BROWNE HCMV INFECTION 2HR UP	6.13E-03
WAMUNYOKOLI OVARIAN CANCER GRADES 1 2 DN	6.13E-03
BIOCARTA IL17 PATHWAY	6.16E-03
GRANDVAUX IRF3 TARGETS DN	6.27E-03
SUNG METASTASIS STROMA UP	6.31E-03
JIANG TIP30 TARGETS UP	6.33E-03
LU AGING BRAIN DN	6.34E-03
SAGIV CD24 TARGETS DN	6.36E-03
KORKOLA TERATOMA	6.36E-03
BERTUCCI MEDULLARY VS DUCTAL BREAST CANCER DN	6.40E-03
RODRIGUES NTN1 AND DCC TARGETS	6.43E-03
WEINMANN ADAPTATION TO HYPOXIA DN	6.43E-03
KIM WT1 TARGETS 12HR UP	6.44E-03
SMID BREAST CANCER LUMINAL A UP	6.45E-03
CHUANG OXIDATIVE STRESS RESPONSE UP	6.73E-03

Table S2 – Continued on next page

Table S2 –continued from previous page

KOYAMA SEMA3B TARGETS UP	6.75E-03
GAUSSMANN MLL AF4 FUSION TARGETS E UP	6.77E-03
BIOCARTA MCALPAIN PATHWAY	6.81E-03
CREIGHTON ENDOCRINE THERAPY RESISTANCE 4	6.86E-03
FARMER BREAST CANCER CLUSTER 5	6.96E-03
BIOCARTA MET PATHWAY	7.00E-03
GARGALOVIC RESPONSE TO OXIDIZED PHOSPHOLIPIDS TURQUOISE UP	7.01E-03
MCBRYAN PUBERTAL BREAST 4 5WK DN	7.04E-03
REACTOME THROMBIN SIGNALLING THROUGH PROTEINASE ACTIVATED RECEPTORS	7.15E-03
YAO TEMPORAL RESPONSE TO PROGESTERONE CLUSTER 0	7.21E-03
BILD HRAS ONCOGENIC SIGNATURE	7.22E-03
BIOCARTA ERK5 PATHWAY	7.28E-03
WOOD EBV EBNA1 TARGETS DN	7.28E-03
WANG ESOPHAGUS CANCER VS NORMAL DN	7.36E-03
YAO TEMPORAL RESPONSE TO PROGESTERONE CLUSTER 1	7.42E-03
ASTIER INTEGRIN SIGNALING	7.48E-03
DAZARD UV RESPONSE CLUSTER G2	7.49E-03
SOTIRIOU BREAST CANCER GRADE 1 VS 3 DN	7.51E-03
REACTOME TRANSMEMBRANE TRANSPORT OF SMALL MOLECULES	7.55E-03
RADMACHER AML PROGNOSIS	7.56E-03
KAAB HEART ATRIUM VS VENTRICLE UP	7.58E-03
ABE INNER EAR	7.82E-03
JI RESPONSE TO FSH DN	7.85E-03
BOQUEST STEM CELL CULTURED VS FRESH DN	7.91E-03
MARTINEZ RB1 AND TP53 TARGETS UP	8.01E-03
GENTILE UV RESPONSE CLUSTER D7	8.08E-03
AMIT SERUM RESPONSE 240 MCF10A	8.27E-03
GENTILE UV RESPONSE CLUSTER D5	8.27E-03
ZHAN MULTIPLE MYELOMA MF UP	8.28E-03
KANG DOXORUBICIN RESISTANCE DN	8.33E-03
WEINMANN ADAPTATION TO HYPOXIA UP	8.35E-03
GRADE COLON AND RECTAL CANCER DN	8.54E-03
REACTOME PLATELET ACTIVATION TRIGGERS	8.54E-03
BIOCARTA TFF PATHWAY	8.54E-03
MOROSETTI FACIOSCAPULOHUMERAL MUSCULAR DISTROPHY UP	8.54E-03
CROONQUIST IL6 DEPRIVATION UP	8.64E-03
KANNAN TP53 TARGETS UP	8.64E-03
WANG SMARCE1 TARGETS UP	8.74E-03

Table S2 – Continued on next page

Table S2 –continued from previous page

KEGG ENDOMETRIAL CANCER	8.85E-03
SENESE HDAC2 TARGETS DN	8.87E-03
REACTOME SEMA4D INDUCED CELL MIGRATION AND GROWTH CONE COLLAPSE	8.88E-03
OSWALD HEMATOPOIETIC STEM CELL IN COLLAGEN GEL UP	8.96E-03
OSWALD HEMATOPOIETIC STEM CELL IN COLLAGEN GEL DN	8.96E-03
REN ALVEOLAR RHABDOMYOSARCOMA DN	8.99E-03
SANCHEZ MDM2 TARGETS	9.07E-03
BIOCARTA RHO PATHWAY	9.17E-03
ST INTEGRIN SIGNALING PATHWAY	9.20E-03
BILD CTNNB1 ONCOGENIC SIGNATURE	9.21E-03
IWANAGA CARCINOGENESIS BY KRAS PTEN DN	9.24E-03
BIOCARTA CDK5 PATHWAY	9.26E-03
HOSHIDA LIVER CANCER LATE RECURRENCE DN	9.36E-03
SCHEIDEREIT IKK TARGETS	9.45E-03
SHEDDEN LUNG CANCER GOOD SURVIVAL A4	9.66E-03
ONDER CDH1 TARGETS 2 UP	9.76E-03
FRIDMAN IMMORTALIZATION DN	9.79E-03
WANG CISPLATIN RESPONSE AND XPC DN	9.84E-03
BROWNE HCMV INFECTION 18HR DN	9.98E-03
REACTOME G PROTEIN BETA GAMMA SIGNALLING	1.00E-02
REACTOME PROSTANOID HORMONES	1.01E-02
TAKAO RESPONSE TO UVB RADIATION DN	1.04E-02
KIM MYC AMPLIFICATION TARGETS UP	1.05E-02
VALK AML WITH EVI1	1.05E-02
VERRECCHIA EARLY RESPONSE TO TGFB1	1.05E-02
WU CELL MIGRATION	1.08E-02
CHANDRAN METASTASIS DN	1.08E-02
HEIDENBLAD AMPLIFIED IN PANCREATIC CANCER	1.11E-02
REACTOME TRKA SIGNALLING FROM THE PLASMA MEMBRANE	1.13E-02
MAHADEVAN IMATINIB RESISTANCE DN	1.14E-02
REACTOME RHO GTPASE CYCLE	1.15E-02
BIOCARTA IL12 PATHWAY	1.16E-02
BROWNE HCMV INFECTION 24HR DN	1.17E-02
KYNG DNA DAMAGE UP	1.19E-02
CERVERA SDHB TARGETS 1 DN	1.19E-02
TRAYNOR RETT SYNDROM UP	1.20E-02
VERRECCHIA RESPONSE TO TGFB1 C3	1.23E-02
BERTUCCI INVASIVE CARCINOMA DUCTAL VS LOBULAR DN	1.24E-02
BILBAN B CLL LPL UP	1.25E-02

Table S2 – Continued on next page

Table S2 –continued from previous page

BROWNE HCMV INFECTION 48HR DN	1.25E-02
DORN ADENOVIRUS INFECTION 48HR UP	1.26E-02
HELLER HDAC TARGETS SILENCED BY METHYLATION UP	1.27E-02
LINDGREN BLADDER CANCER CLUSTER 2A DN	1.28E-02
AIYAR COBRA1 TARGETS UP	1.28E-02
GENTILE UV RESPONSE CLUSTER D2	1.29E-02
DORN ADENOVIRUS INFECTION 12HR DN	1.31E-02
ACEVEDO LIVER TUMOR VS NORMAL ADJACENT TISSUE DN	1.31E-02
GAUSSMANN MLL AF4 FUSION TARGETS F UP	1.31E-02
SWEET KRAS ONCOGENIC SIGNATURE	1.32E-02
CHARAFE BREAST CANCER LUMINAL VS BASAL DN	1.33E-02
SENESE HDAC3 TARGETS UP	1.33E-02
KEGG GAP JUNCTION	1.33E-02
KOKKINAKIS METHIONINE DEPRIVATION 96HR UP	1.33E-02
DUNNE TARGETS OF AML1 MTG8 FUSION DN	1.35E-02
HUANG DASATINIB RESISTANCE UP	1.36E-02
REACTOME SEMA3A PLEXIN REPULSION SIGNALING BY INHIBITING INTEGRIN ADHESION	1.37E-02
BIOCARTA IL6 PATHWAY	1.37E-02
GENTILE UV HIGH DOSE DN	1.38E-02
MARTINEZ TP53 TARGETS UP	1.39E-02
MEINHOLD OVARIAN CANCER LOW GRADE UP	1.39E-02
REACTOME ACTIVATION OF KAINATE RECEPTORS UPON GLUTAMATE BINDING	1.40E-02
BIOCARTA NO2IL12 PATHWAY	1.40E-02
NAKAMURA METASTASIS	1.42E-02
BIOCARTA PTEN PATHWAY	1.42E-02
BIOCARTA ECM PATHWAY	1.43E-02
BIOCARTA CDC42RAC PATHWAY	1.45E-02
SESTO RESPONSE TO UV C5	1.47E-02
CHARAFE BREAST CANCER BASAL VS MESENCHYMAL UP	1.47E-02
DANG REGULATED BY MYC DN	1.48E-02
NIKOLSKY BREAST CANCER 6P24 P22 AMPLICON	1.49E-02
EHRLICH ICF SYNDROM UP	1.49E-02
ACEVEDO LIVER CANCER DN	1.49E-02
BONOME OVARIAN CANCER POOR SURVIVAL UP	1.52E-02

5 GSVA for RNA-Seq data

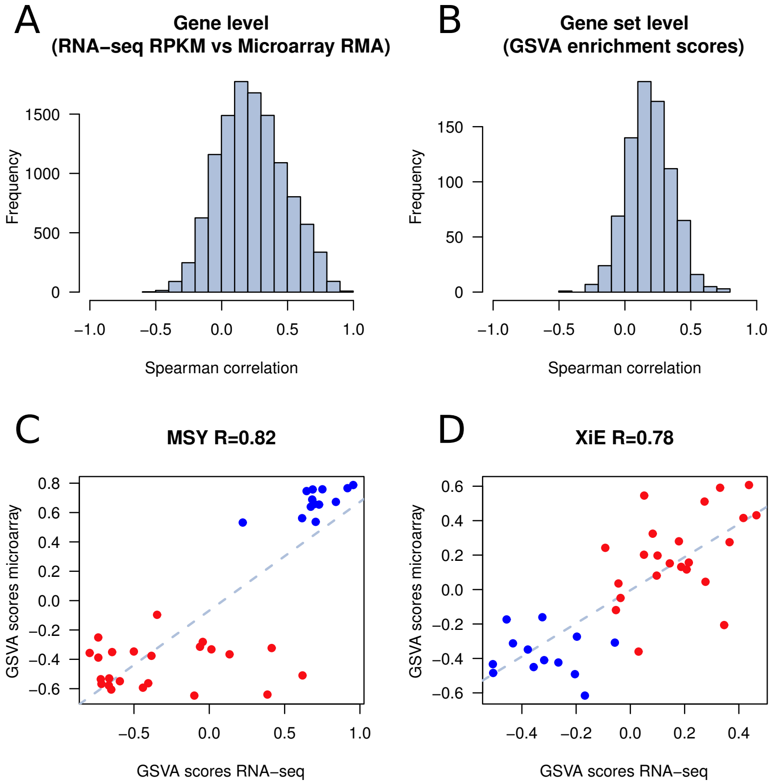

Figure S4: **GSVA for RNA-seq (Yale).** A. Distribution of Spearman correlation values between gene expression profiles of RNA-seq and microarray data. B. Distribution of Spearman correlation values between GSVA enrichment scores of gene sets calculated from RNA-seq and microarray data. C and D. Comparison of GSVA enrichment scores obtained from microarray and RNA-seq data for two gene sets containing genes with sex-specific expression: MSY formed by genes from the male-specific region of the Y chromosome (male-specific), and XiE formed by genes that escape X-inactivation in females (female-specific). Red and blue dots represent female and male samples, respectively. In both cases GSVA scores show very high correlation between the two profiling technologies where female samples show higher enrichment scores in the female-specific gene set and male samples show higher enrichment scores in the male-specific gene set.

6 CNV Analysis in Ovarian Data

GSVA enables comparisons with different molecular data types, such as measurements of copy-number variation (CNV). Our approach is analogous to eQTL analysis, which studies how genetic variation in DNA impacts changes in mRNA expression. We would like to discern how copy number alterations, induced by either deletions or amplifications along a chromosomal region, produce changes in pathway activity. For this, we use the ovarian cancer data (OV) from TCGA.

We begin by preprocessing the CNV data. First, we remove any *cis*-CNV effect on mRNA expression since we are not interested in pathways whose behavior can be explained by occupying a shared genomic region. For this, we fit a linear model between gene expression and the CNV overlapping a gene, and regress out the CNV's effect on expression. CNV data was further reduced in dimension by windowing the data into 60kb blocks, with 30kb steps across each chromosome, resulting in approximately 11,000 loci.

We use this corrected expression data to compute GSVA enrichment scores using the maximum deviation method, followed by CNV-pathway correlation analysis. We separately analyze two gene set databases from MSigDB, the "canonical pathway" gene sets ($n = 833$) (comprised of REACTOME, BIOCARTA and KEGG) and the gene ontology (GO) biological process gene sets ($n = 885$), and plot the number of gene sets with a strong correlation ($|\rho| > .4$) at a given locus (Figure S5). The canonical pathways track, shown in light gray, has its largest peak on the p-arm of chromosome 6 and corresponds primarily to immune related pathways, a likely consequence of major histocompatibility complex (MHC) genes found densely in this region. The highest ranked CNV-pathway correlations ($|\rho| > .8$) occur at three loci on chr4:68MB, chr6:26MB, and chr6:32MB corresponding to pathways *KEGG PENTOSE AND GLUCURONATE INTERCONVERSIONS*, *REACTOME RNA POLYMERASE I PROMOTER OPENING*, and *KEGG ANTIGEN PROCESSING AND PRESENTATION*, respectively. The first of these gene sets is particularly interesting, as it may relate to the so-called "Warburg effect" seen in cancer tumors whereby anaerobic glycolysis is increased to supply fast growing tumors with needed energy. The GO gene sets track is depicted in dark gray and shows a large peak on chromosome 17. This region consists of pathways primarily associated with viral response and cation homeostasis. Only one CNV region has a gene set with a correlation above the $\rho = |.8|$ threshold: *EXTRACELLULAR STRUCTURE ORGANIZATION AND BIOGENESIS* at the region chr5:138MB - chr5:143MB.

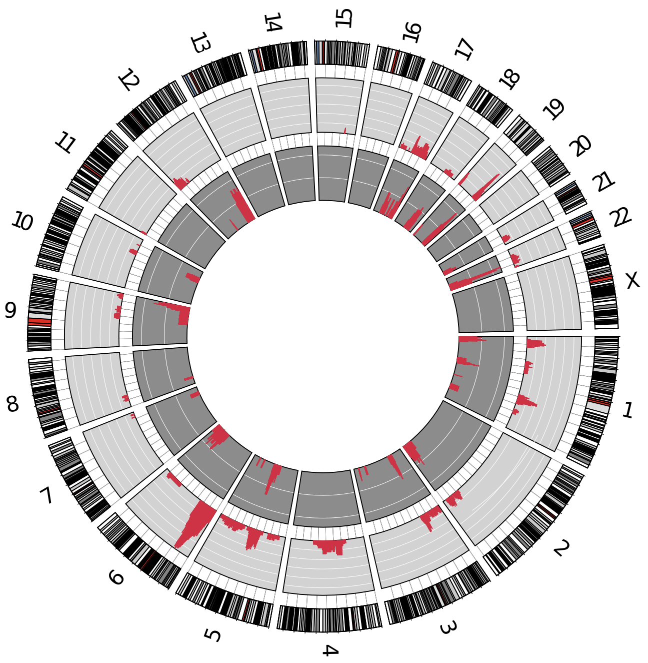

Figure S5: **CNV-pathway correlation analysis.** Depicted in red are the correlations of GSVA scores with windowed CNV for the canonical pathways (light gray track) and the GO terms (dark gray track). Only correlations $|\rho| > .4$ are shown at each locus.

References

[1] Gordon K Smyth. Linear models and empirical bayes methods for assessing differential expression in microarray experiments. *Statistical Applications in Genetics and Molecular Biology*, 3(1), 2004.

[2] Thomas J Giordano, Rork Kuick, Tobias Else, Paul G Gauger, Michelle Vinco, Juliane Bauersfeld, Donita Sanders, Dafydd G Thomas, Gerard Doherty, and Gary Hammer. Molecular classification and prognostication of adrenocortical tumors by transcriptome profiling. *Clinical Cancer Research: An Official Journal of the American Association for Cancer Research*, 15(2):668–676, January 2009. PMID: 19147773.

[3] Martin Fassnacht, Rossella Libé, Matthias Kroiss, and Bruno Allolio. Adrenocortical carcinoma: a clinician's update. *Nature Reviews. Endocrinology*, March 2011. PMID: 21386792.

[4] Rossella Libe, Amato Fratticci, and Jerome Bertherat. Adrenocortical cancer: pathophysiology and clinical management. *Endocr Relat Cancer*, 14(1):13–28, March 2007.

[5] Bruno Ragazzon, Guillaume Assie, and Jerome Bertherat. Transcriptome analysis of adrenocortical cancers: from molecular classification to the identification of new treatments. *Endocr Relat Cancer*, 18(2):R15–27, February 2011.

[6] Xiao-cao Shen, Cai-xiao Gu, Yi-qing Qiu, Chuan-jun Du, Yan-biao Fu, and Jian-jun Wu. Estrogen receptor expression in adrenocortical carcinoma. *Journal of Zhejiang University. Science. B*, 10(1):1–6, January 2009. PMID: 19198016 PMCID: 2613956.

[7] Gary D. Hammer and Tobias Else, editors. *Adrenocortical Carcinoma*. Springer New York, New York, NY, 2011.

[8] Aurélien de Reyniès, Guillaume Assié, David S. Rickman, Frédérique Tissier, Lionel Groussin, Fernande René-Corail, Bertrand Dousset, Xavier Bertagna, Eric Clauser, and Jérôme Bertherat. Gene expression profiling reveals a new classification of adrenocortical tumors and identifies molecular predictors of malignancy and survival. *Journal of Clinical Oncology*, 27(7):1108 –1115, March 2009.

[9] Jean J. Zhao, Thomas M. Roberts, and William C. Hahn. Functional genetics and experimental models of human cancer. *Trends in Molecular Medicine*, 10(7):344–350, July 2004.
